# Complete Genome Sequence of *Bacillus megaterium* Podophage Pavlov

**DOI:** 10.1128/genomeA.00993-15

**Published:** 2015-09-03

**Authors:** Roberto W. Burgos, Scott J. Mash, Jesse L. Cahill, Eric S. Rasche, Gabriel F. Kuty Everett

**Affiliations:** Center for Phage Technology, Texas A&M University, College Station, Texas, USA

## Abstract

*Bacillus megaterium* is a large spore-forming bacterium found widely in the environment. Phages infecting *B. megaterium* can be used as genetic tools to expand the array of uses of *B. megaterium* in research and industry. Here, we present the complete genome of Pavlov, a podophage infecting *B. megaterium.*

## GENOME ANNOUNCEMENT

*Bacillus megaterium* is a well-studied model organism in the field of microbiology. Due to its large size, secretion ability, and stable plasmid replication system, it has become a workhorse for recombinant protein production in the biotechnology industry (1). Recently, the systems metabolic engineering of *B. megaterium* has gained interest (2). Bacteriophages have a broad array of applications (3) and may play a role in developing the full potential of industrial *B. megaterium* strains. To that end, here we describe the novel *B. megaterium* podophage Pavlov.

Bacteriophage Pavlov was isolated from a soil sample collected in College Station, Texas, USA, based on its ability to grow on the asporogenic strain *B. megaterium* KM (ATCC 13632). Phage DNA was sequenced in an Illumina MiSeq 250-bp paired-end run with a 550-bp insert library at the Genomic Sequencing and Analysis Facility at the University of Texas (Austin, TX, USA). Quality-controlled, trimmed reads were assembled to a single contig of circular assembly at 25.4-fold coverage using SPAdes version 3.5.0. The contig was confirmed to be complete by PCR using primers that face the upstream and downstream ends of the contig. Products from the PCR amplification of the junctions of concatemeric molecules were sequenced by Sanger sequencing (Eton Bioscience, San Diego, CA, USA). Genes were predicted using GeneMarkS (4) and corrected using software tools available on the Center for Phage Technology (CPT) Galaxy instance (https://cpt.tamu.edu/galaxy-pub/). The morphology of Pavlov was determined using transmission electron microscopy performed at the Texas A&M University Microscopy and Imaging Center.

Pavlov is a podophage with a 40-kb genome, a coding density of 96.2%, and a G+C content of 40.6%. Genome analysis and annotation shows 50 coding sequences, of which 22 have a predicted function by BLASTp and InterPro Scan (5, 6). Pavlov shares 89.0, 91.9, 91.0, 87.1, and 67.6% nucleotide sequence identity with recently described *B. megaterium* podophages Page (NCBI reference sequence NC_022764) (7), Pony (NCBI reference sequence NC_022770) (8), Pookie (GenBank accession number KM236248) (9), Palmer (KP411017) (10), and Pascal (KM236247) (11) as determined by Emboss Stretcher (12). Pavlov is predicted to use a *pac*-type head-full DNA packaging mechanism and has been opened to the *terS* gene for annotation purposes (13). Host range studies show that Pavlov also infects the industrial *B. megaterium* strains PV361 and DSM 337 (1).

Replication, biosynthesis, packaging, transcriptional regulation, morphogenesis, lysis genes, and an HNH-homing endonuclease were identified in Pavlov. Three DNA-binding proteins, presumably transcriptional regulators, were found to contain lambda Cro/CI-type helix-turn-helix domains. Additionally, the presence of a plasmid replication/relaxation protein suggests that Pavlov may be a temperate phage. Like other phages of this group, Pavlov encodes an FtsK/SpoIIIE homolog. In Gram-positive bacteria, SpoIIIE is an ATPase that translocates DNA across the septal membrane of the sporulating mother cell into the forespore, although its role in the phage life cycle is unknown (14). Pavlov encodes a SigF-like sporulation-related sigma factor, indicating that the phage might influence the vegetative growth and sporulation of the host (15).

### Nucleotide sequence accession number. 

The genome sequence of phage Pavlov was contributed to GenBank under the accession number KT001911.
